# A scoping review of the literature on the current mental health status of physicians and physicians-in-training in North America

**DOI:** 10.1186/s12889-019-7661-9

**Published:** 2019-10-24

**Authors:** Mara Mihailescu, Elena Neiterman

**Affiliations:** 1 Telfer School of Management, University of Ottawa, 55 Laurier Ave E, Ottawa, ON K1N 6N5 Canada; 2 School of Public Health and Health Systems, University of Waterloo, 200 University Avenue West, Waterloo, ON N2L 3G1 Canada

**Keywords:** Mental health, Burnout, Mental illness, Physicians, Medical students, Residents, Scoping review, Interventions, Barriers, North America

## Abstract

**Background:**

This scoping review summarizes the existing literature regarding the mental health of physicians and physicians-in-training and explores what types of mental health concerns are discussed in the literature, what is their prevalence among physicians, what are the causes of mental health concerns in physicians, what effects mental health concerns have on physicians and their patients, what interventions can be used to address them, and what are the barriers to seeking and providing care for physicians. This review aims to improve the understanding of physicians’ mental health, identify gaps in research, and propose evidence-based solutions.

**Methods:**

A scoping review of the literature was conducted using Arksey and O’Malley’s framework, which examined peer-reviewed articles published in English during 2008–2018 with a focus on North America. Data were summarized quantitatively and thematically.

**Results:**

A total of 91 articles meeting eligibility criteria were reviewed. Most of the literature was specific to burnout (*n* = 69), followed by depression and suicidal ideation (*n* = 28), psychological harm and distress (*n* = 9), wellbeing and wellness (*n* = 8), and general mental health (*n* = 3). The literature had a strong focus on interventions, but had less to say about barriers for seeking help and the effects of mental health concerns among physicians on patient care.

**Conclusions:**

More research is needed to examine a broader variety of mental health concerns in physicians and to explore barriers to seeking care. The implication of poor physician mental health on patients should also be examined more closely. Finally, the reviewed literature lacks intersectional and longitudinal studies, as well as evaluations of interventions offered to improve mental wellbeing of physicians.

## Background

The World Health Organization (WHO) defines mental health as “a state of well-being in which the individual realizes his or her own abilities, can cope with the normal stresses of life, can work productively and fruitfully, and is able to make a contribution to his or her community.” [41] One in four people worldwide are affected by mental health concerns [40]. Physicians are particularly vulnerable to experiencing mental illness due to the nature of their work, which is often stressful and characterized by shift work, irregular work hours, and a high pressure environment [1, 21, 31]. In North America, many physicians work in private practices with no access to formal institutional supports, which can result in higher instances of social isolation [13, 27]. The literature on physicians’ mental health is growing, partly due to general concerns about mental wellbeing of health care workers and partly due to recognition that health care workers globally are dissatisfied with their work, which results in burnout and attrition from the workforce [31, 34]. As a consequence, more efforts have been made globally to improve physicians’ mental health and wellness, which is known as “The Quadruple Aim.” [34] While the literature on mental health is flourishing, however, it has not been systematically summarized. This makes it challenging to identify what is being done to improve physicians’ wellbeing and which solutions are particularly promising [7, 31, 33, 37, 38]. The goal of our paper is to address this gap.

This paper explores what is known from the existing peer-reviewed literature about the mental health status of physicians and physicians-in-training in North America. Specifically, we examine (1) what types of mental health concerns among physicians are commonly discussed in the literature; (2) what are the reported causes of mental health concerns in physicians; (3) what are the effects that mental health concerns may have on physicians and their patients; (4) what solutions are proposed to improve mental health of physicians; and (5) what are the barriers to seeking and providing care to physicians with mental health concerns. Conducting this scoping review, our goal is to summarize the existing research, identifying the need for a subsequent systematic review of the literature in one or more areas under the study. We also hope to identify evidence-based interventions that can be utilized to improve physicians’ mental wellbeing and to suggest directions for future research [2]. Evidence-based interventions might have a positive impact on physicians and improve the quality of patient care they provide.

## Methods

A scoping review of the academic literature on the mental health of physicians and physicians-in-training in North America was conducted using Arksey and O’Malley’s [2] methodological framework. Our review objectives and broad focus, including the general questions posed to conduct the review, lend themselves to a scoping review approach, which is suitable for the analysis of a broader range of study designs and methodologies [2]. Our goal was to map the existing research on this topic and identify knowledge gaps, without making any prior assumptions about the literature’s scope, range, and key findings [29].

### Stage 1: identify the research question

Following the guidelines for scoping reviews [2], we developed a broad research question for our literature search, asking *what does the academic literature tell about mental health issues among physicians, residents, and medical students in North America*? Burnout and other mental health concerns often begin in medical training and continue to worsen throughout the years of practice [31]. Recognizing that the study and practice of medicine plays a role in the emergence of mental health concerns, we focus on practicing physicians – general practitioners, specialists, and surgeons – and those who are still in training – residents and medical students. We narrowed down the focus of inquiry by asking the following sub-questions:

1. What types of mental health concerns among physicians are commonly discussed in the literature?
2. What are the reported causes of mental health problems in physicians and what solutions are available to improve the mental wellbeing of physicians?
3. What are the barriers to seeking and providing care to physicians suffering from mental health problems?

### Stage 2: identify the relevant studies

We included in our review empirical papers published during January 2008–January 2018 in peer-reviewed journals. Our exclusive focus on peer-reviewed and empirical literature reflected our goal to develop an evidence-based platform for understanding mental health concerns in physicians. Since our focus was on prevalence of mental health concerns and promising practices available to physicians in North America, we excluded articles that were more than 10 years old, suspecting that they might be too outdated for our research interest. We also excluded papers that were not in English or outside the region of interest. Using combinations of keywords developed in consultation with a professional librarian (See Table 1), we searched databases PUBMed, SCOPUS, CINAHL, and PsychNET. We also screened reference lists of the papers that came up in our original search to ensure that we did not miss any relevant literature.

**Table 1:** Terms used for the literature search

(physicians/psychology [mesh]) AND (burnout, professional OR mental health OR depression OR anxiety OR exhaustion) AND (policy OR programs or policies OR best practices*[ti])	
(residents/psychology [mesh]) AND (burnout, professional OR mental health OR depression OR anxiety OR exhaustion) AND (policy OR programs or policies OR best practices*[ti])	
(students, medical/psychology [mesh]) AND (burnout, professional OR mental health OR depression OR anxiety OR exhaustion) AND (policy OR programs or policies OR best practices*[ti])	

### Stage 3: literature selection

**Fig. 1:**
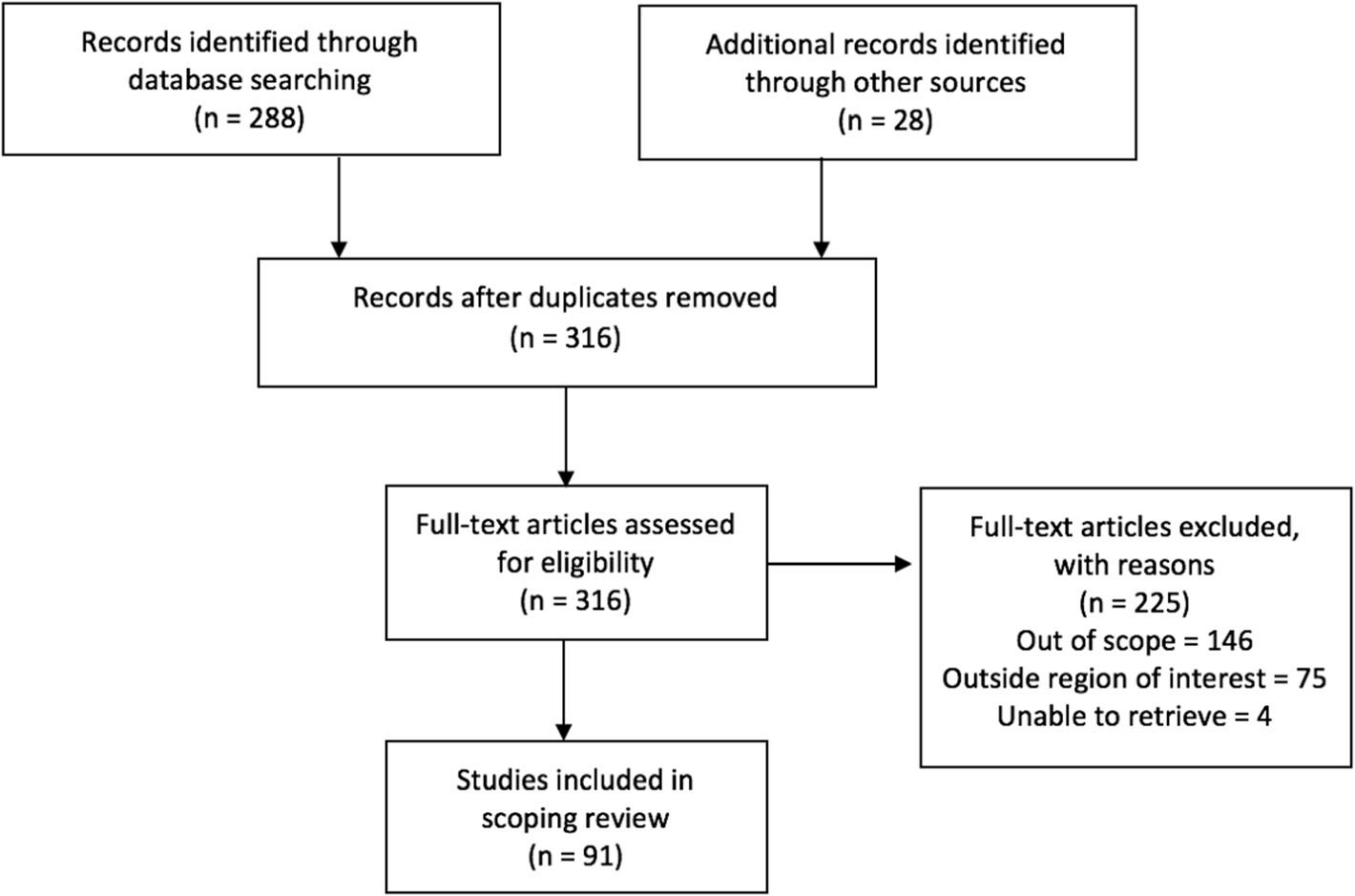
PRISMA Flow Diagram

Publications were imported into a reference manager and screened for eligibility. During initial abstract screening, 146 records were excluded for being out of scope, 75 records were excluded for being outside the region of interest, and 4 papers were excluded because they could not be retrieved. The remaining 91 papers were included into the review. Figure 1 summarizes the literature search and selection.

### Stage 4: charting the data

A literature extraction tool was created in Microsoft Excel to record the author, date of publication, location, level of training, type of article (empirical, report, commentary), and topic. Both authors coded the data inductively, first independently reading five articles and generating themes from the data, then discussing our coding and developing a coding scheme that was subsequently applied to ten more papers. We then refined and finalized the coding scheme and used it to code the rest of the data. When faced with disagreements on narrowing down the themes, we discussed our reasoning and reached consensus.

### Stage 5: collating, summarizing, and reporting the results

The data was summarized by frequency and type of publication, mental health topics, and level of training. The themes inductively derived from the data included (1) description of mental health concerns affecting physicians and physicians-in-training; (2) prevalence of mental health concerns among this population; (3) possible causes that can explain the emergence of mental health concerns; (4) solutions or interventions proposed to address mental health concerns; (5) effects of mental health concerns on physicians and on patient outcomes; and (6) barriers for seeking and providing help to physicians afflicted with mental health concerns. Each paper was coded based on its relevance to major theme(s) and, if warranted, secondary focus. Therefore, one paper could have been coded in more than one category. Upon analysis, we identified the gaps in the literature.

## Results

### Characteristics of included literature

**Fig. 2:**
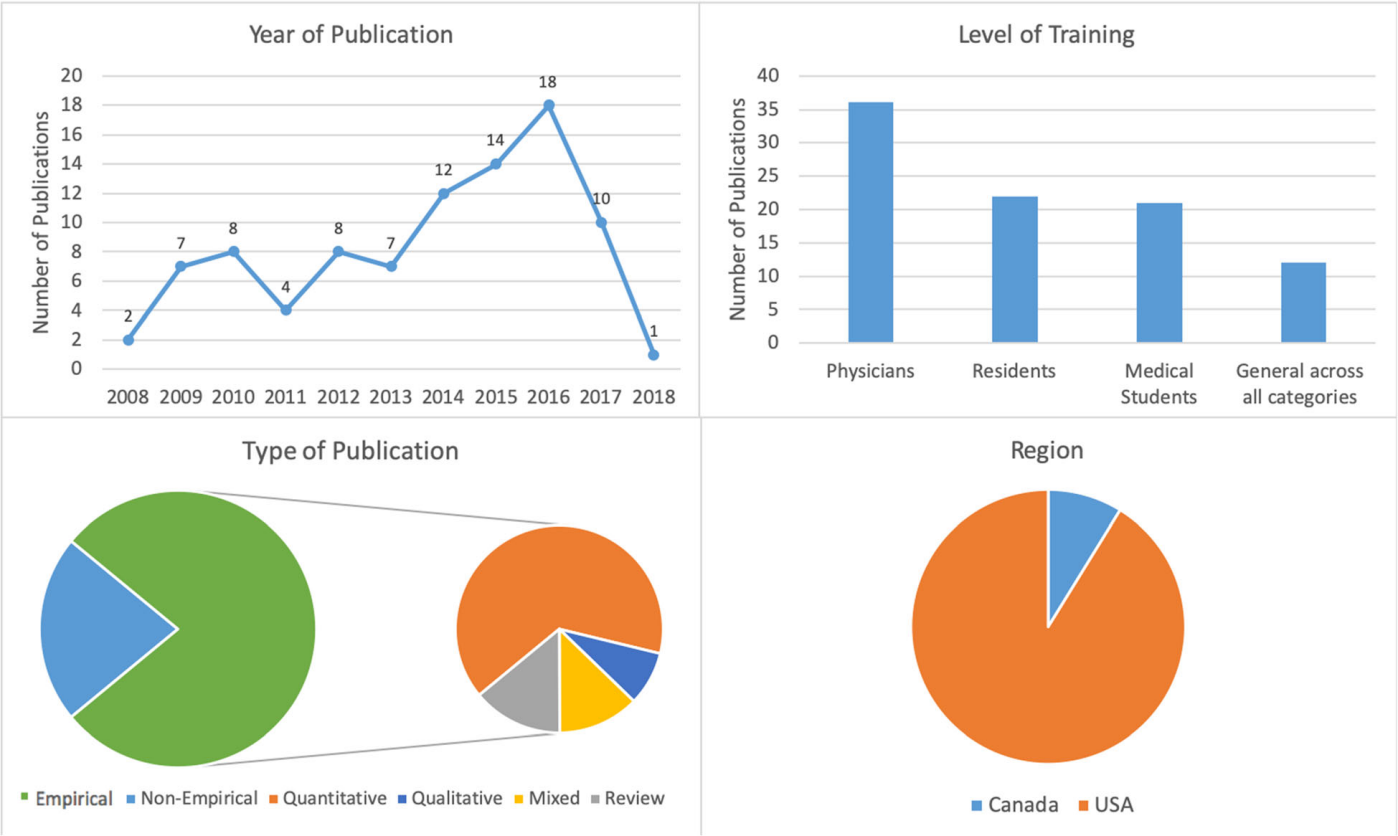
Number of sources by characteristics of included literature

**Fig. 3:**
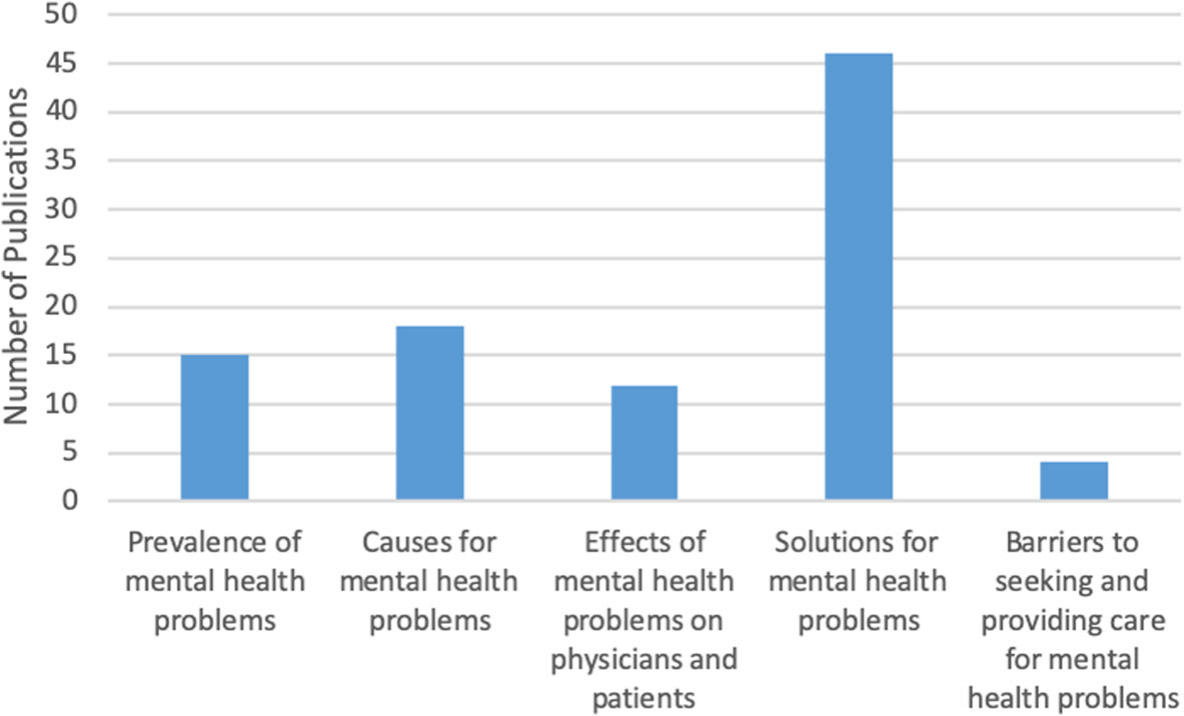
Frequency of themes in literature (*n* = 91)

The initial search yielded 316 records of which 91 publications underwent full-text review and were included in our scoping review. Our analysis revealed that the publications appear to follow a trend of increase over the course of the last decade reflecting the growing interest in physicians’ mental health. More than half of the literature was published in the last 4 years included in the review, from 2014 to 2018 (*n* = 55), with most publications in 2016 (*n* = 18) (Fig. 2). The majority of papers (*n* = 36) focused on practicing physicians, followed by papers on residents (*n* = 22), medical students (*n* = 21), and those discussing medical professionals with different level of training (*n* = 12). The types of publications were mostly empirical (*n* = 71), of which 46 papers were quantitative. Furthermore, the vast majority of papers focused on the United States of America (USA) (*n* = 83), with less than 9% focusing on Canada (*n* = 8). The frequency of identified themes in the literature is broken down into prevalence of mental health concerns (*n* = 15), causes of mental health concerns (*n* = 18), effects of mental health concerns on physicians and patients (*n* = 12), solutions and interventions for mental health concerns (*n* = 46), and barriers to seeking and providing care for mental health concerns (*n* = 4) (Fig. 3).

### Mental health concerns and their prevalence in the literature

**Fig. 4:**
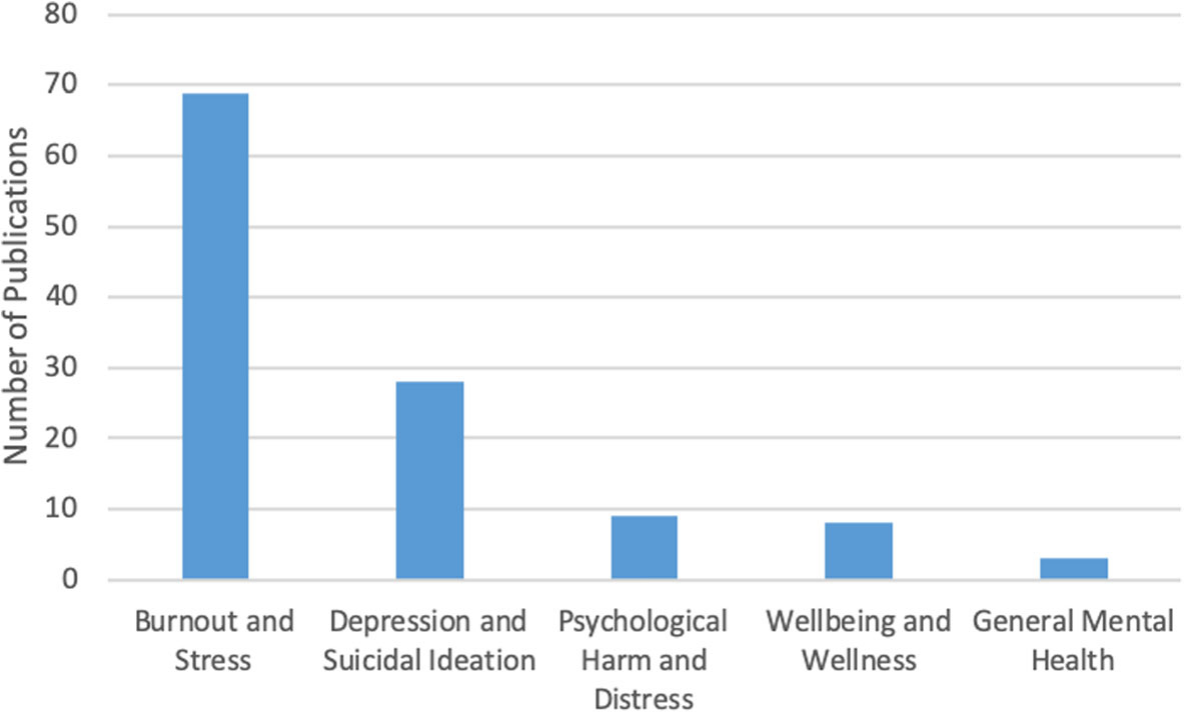
Number of sources by mental health topic discussed (*n* = 91)

In this thematic category (*n* = 15), we coded the papers discussing the prevalence of specific mental health concerns among physicians and those comparing physicians’ mental health to that of the general population. Most papers focused on burnout and stress (*n* = 69), which was followed by depression and suicidal ideation (*n* = 28), psychological harm and distress (*n* = 9), wellbeing and wellness (*n* = 8), and general mental health (*n* = 3) (Fig. 4). The literature also identified that, on average, burnout and mental health concerns affect 30–60% of all physicians and residents [4, 5, 8, 9, 15, 25, 26].

There was some overlap between the papers discussing burnout, depression, and suicidal ideation, suggesting that work-related stress may lead to the emergence of more serious mental health problems [3, 12, 21], as well as addiction and substance abuse [22, 27]. Residency training was shown to produce the highest rates of burnout [4, 8, 19].

### Causes of mental health concerns

Papers discussing the causes of mental health concerns in physicians formed the second largest thematic category (*n* = 18). Unbalanced schedules and increasing administrative work were defined as key factors in producing poor mental health among physicians [4–6, 13, 15, 27]. Some papers also suggested that the nature of the medical profession itself – competitive culture and prioritizing others – can lead to the emergence of mental health concerns [23, 27]. Indeed, focus on qualities such as rigidity, perfectionism, and excessive devotion to work during the admission into medical programs fosters the selection of students who may be particularly vulnerable to mental illness in the future [21, 24]. The third cluster of factors affecting mental health stemmed from structural issues, such as pressure from the government and insurance, fragmentation of care, and budget cuts [13, 15, 18]. Work overload, lack of control over work environment, lack of balance between effort and reward, poor sense of community among staff, lack of fairness and transparency by decision makers, and dissonance between one’s personal values and work tasks are the key causes for mental health concerns among physicians [20]. Govardhan et al. conceptualized causes for mental illness as having a cyclical nature - depression leads to burnout and depersonalization, which leads to patient dissatisfaction, causing job dissatisfaction and more depression [19].

### Effects of mental health concerns on physicians and patients

A relatively small proportion of papers (13%) discussed the effects of mental health concerns on physicians and patients. The literature prioritized the direct effect of mental health on physicians (*n* = 11) with only one paper focusing solely on the indirect effects physicians’ mental health may have on patients. Poor mental health in physicians was linked to decreased mental and physical health [3, 14, 15]. In addition, mental health concerns in physicians were associated with reduction in work hours and the number of patients seen, decrease in job satisfaction, early retirement, and problems in personal life [3, 5, 15]. Lu et al. found that poor mental health in physicians may result in increased medical errors and the provision of suboptimal care [25]. Thus physicians’ mental wellbeing is linked to the quality of care provided to patients [3–5, 10, 17].

### Solutions and interventions

In this largest thematic category (*n* = 46) we coded the literature that offered solutions for improving mental health among physicians. We identified four major levels of interventions suggested in the literature. A sizeable proportion of literature discussed the interventions that can be broadly categorized as *primary prevention* of mental illness. These papers proposed to increase awareness of physicians’ mental health and to develop strategies that can help to prevent burnout from occurring in the first place [4, 12]. Some literature also suggested programs that can help to increase resilience among physicians to withstand stress and burnout [9, 20, 27]. We considered the papers referring to the strategies targeting physicians currently suffering from poor mental health as *tertiary prevention*. This literature offered insights about mindfulness-based training and similar wellness programs that can increase self-awareness [ 16, 18, 27], as well as programs aiming to improve mental wellbeing by focusing on physical health [17].

While the aforementioned interventions target individual physicians, some literature proposed *workplace/institutional* interventions with primary focus on changing workplace policies and organizational culture [4, 13, 23, 25]. Reducing hours spent at work and paperwork demands or developing guidelines for how long each patient is seen have been identified by some researchers as useful strategies for improving mental health [6, 11, 17]. Offering access to mental health services outside of one’s place of employment or training could reduce the fear of stigmatization at the workplace [5, 12]. The proposals for cultural shift in medicine were mainly focused on promoting a less competitive culture, changing power dynamics between physicians and physicians-in-training, and improving wellbeing among medical students and residents. The literature also proposed that the medical profession needs to put more emphasis on supporting trainees, eliminating harassment, and building strong leadership [23]. Changing curriculum for medical students was considered a necessary step for the cultural shift [20]. Finally, while we only reviewed one paper that directly dealt with the *governmental level* of prevention, we felt that it necessitated its own sub-thematic category because it identified the link between government policy, such as health care reforms and budget cuts, and the services and care physicians can provide to their patients [13].

### Barriers to seeking and providing care

Only four papers were summarized in this thematic category that explored what the literature says about barriers for seeking and providing care for physicians suffering from mental health concerns. Based on our analysis, we identified two levels of factors that can impact access to mental health care among physicians and physicians-in-training.

*Individual level barriers* stem from intrinsic barriers that individual physicians may experience, such as minimizing the illness [21], refusing to seek help or take part in wellness programs [14], and promoting the culture of stoicism [27] among physicians. Another barrier is stigma associated with having a mental illness. Although stigma might be experienced personally, literature suggests that acknowledging the existence of mental health concerns may have negative consequences for physicians, including loss of medical license, hospital privileges, or professional advancement [10, 21, 27].

*Structural barriers* refer to the lack of formal support for mental wellbeing [3], poor access to counselling [6], lack of promotion of available wellness programs [10], and cost of treatment. Lack of research that tests the efficacy of programs and interventions aiming to improve mental health of physicians makes it challenging to develop evidence-based programs that can be implemented at a wider scale [5, 11, 12, 18, 20].

## Discussion

Our analysis of the existing literature on mental health concerns in physicians and physicians-in-training in North America generated five thematic categories. Over half of the reviewed papers focused on proposing solutions, but only a few described programs that were empirically tested and proven to work. Less common were papers discussing causes for deterioration of mental health in physicians (20%) and prevalence of mental illness (16%). The literature on the effects of mental health concerns on physicians and patients (13%) focused predominantly on physicians with only a few linking physicians’ poor mental health to medical errors and decreased patient satisfaction [3, 4, 16, 24]. We found that the focus on barriers for seeking and receiving help for mental health concerns (4%) was least prevalent. The topic of burnout dominated the literature (76%). It seems that the nature of physicians’ work fosters the environment that causes poor mental health [1, 21, 31].

While emphasis on burnout is certainly warranted, it might take away the attention paid to other mental health concerns that carry more stigma, such as depression or anxiety. Establishing a more explicit focus on other mental health concerns might promote awareness of these problems in physicians and reduce the fear such diagnosis may have for doctors’ job security [10]. On the other hand, utilizing the popularity and non-stigmatizing image of “burnout” might be instrumental in developing interventions promoting mental wellbeing among a broad range of physicians and physicians-in-training.

Table 2 summarizes the key findings from the reviewed literature that are important for our understanding of physician mental health. In order to explicitly summarize the gaps in the literature, we mapped them alongside the areas that have been relatively well studied. We found that although non-empirical papers discussed physicians’ mental wellbeing broadly, most empirical papers focused on medical specialty (e.g. neurosurgeons, family medicine, etc.) [4, 8, 15, 19, 25, 28, 35, 36]. Exclusive focus on professional specialty is justified if it features a unique context for generation of mental health concerns, but it limits the ability to generalize the findings to a broader population of physicians. Also, while some papers examined the impact of gender on mental health [7, 32, 39], only one paper considered ethnicity as a potential factor for mental health concerns and found no association [4]. Given that mental health in the general population varies by gender, ethnicity, age, and sexual orientation, it would be prudent to examine mental health among physicians using an intersectional analysis [30, 32, 39]. Finally, of the empirical studies we reviewed, all but one had a cross-sectional design. Longitudinal design might offer a better understanding of the emergence and development of mental health concerns in physicians and tailor interventions to different stages of professional career. Additionally, it could provide an opportunity to evaluate programs’ and policies’ effectiveness in improving physicians’ mental health. This would also help to address the gap that we identified in the literature – an overarching focus on proposing solutions with little demonstrated evidence they actually work.

**Table 2:** Knowledge and gaps in the literature on mental health of physicians in North America

Current focus in the literature	Gaps identified
We have a solid knowledge on mental health status of practicing physicians	We need to learn more about the role curricula and program changes have on medical students’ and medical residents’ mental health
Most empirical research focuses on a particular medical specialty	We need more research on the mental health status of physicians across different specialties
A substantial amount of literature deals with burnout	More research is needed on common mental health concerns, such as depression, anxiety and other conditions that can be perceived as more stigmatizing
Some studies identify the link between gender and mental health concerns in physicians	We need more intersectional analyses, including interactions of medical practice with gender, ethnicity, age, and sexual orientation
Most of the existing empirical research is cross-sectional	We need more longitudinal studies
Literature proposes a variety of programs and interventions to improve mental wellbeing among physicians	We need more research aimed to test the effectiveness of the proposed programs and interventions
We know about the impact that mental health concerns have on physicians’ personal and professional life	More research is needed to explore how physicians’ mental wellbeing can enhance quality of care provided to patients

This review has several limitations. First, our focus on academic literature may have resulted in overlooking the papers that are not peer-reviewed but may provide interesting solutions to physician mental health concerns. It is possible that grey literature – reports and analyses published by government and professional organizations – offers possible solutions that we did not include in our analysis or offers a different view on physicians’ mental health. Additionally, older papers and papers not published in English may have information or interesting solutions that we did not include in our review. Second, although our findings suggest that the theme of burnout dominated the literature, this may be the result of the search criteria we employed. Third, following the scoping review methodology [2], we did not assess the quality of the papers, focusing instead on the overview of the literature. Finally, our research was restricted to North America, specifically Canada and the USA. We excluded Mexico because we believed that compared to the context of medical practice in Canada and the USA, which have some similarities, the work experiences of Mexican physicians might be different and the proposed solutions might not be readily applicable to the context of practice in Canada and the USA. However, it is important to note that differences in organization of medical practice in Canada and the USA do exist, as do differences across and within provinces in Canada and the USA. A comparative analysis can shed light on how the structure and organization of medical practice shapes the emergence of mental health concerns.

## Conclusions

The scoping review we conducted contributes to the existing research on mental wellbeing of American and Canadian physicians by summarizing key knowledge areas and identifying key gaps and directions for future research. While the papers reviewed in our analysis focused on North America, we believe that they might be applicable to the global medical workforce. Identifying key gaps in our knowledge, we are calling for further research on these topics, including examination of medical training curricula and its impact on mental wellbeing of medical students and residents, research on common mental health concerns such as depression or anxiety, studies utilizing intersectional and longitudinal approaches, and program evaluations assessing the effectiveness of interventions aiming to improve mental wellbeing of physicians. Focus on the effect physicians’ mental health may have on the quality of care provided to patients might facilitate support from government and policy makers. We believe that large-scale interventions that are proven to work effectively can utilize an upstream approach for improving the mental health of physicians and physicians-in-training.

## Data Availability

The datasets used and/or analyzed during the current study are available from the corresponding author on reasonable request.

## Abbreviations

PRISMA: Preferred Reporting Items for Systematic Reviews and Meta-Analyses
USA: United States of America
WHO: World Health Organization
